# Chronic whiplash and central sensitization; an evaluation of the role of a myofascial trigger points in pain modulation

**DOI:** 10.1186/1749-7221-4-2

**Published:** 2009-04-23

**Authors:** Michael D Freeman, Ake Nystrom, Christopher Centeno

**Affiliations:** 1 Department of Public Health and Preventive Medicine, Oregon Health and Science University School of Medicine, Portland, Oregon, USA; 2 Institute of Forensic Medicine, Faculty of Health Sciences, University of Aarhus, Aarhus, Denmark; 3 University of Nebraska Medical Center, Omaha, Nebraska, USA; 4 Spinal Injury Foundation, Westminster, Colorado, USA

## Abstract

**Objective:**

it has been established that chronic neck pain following whiplash is associated with the phenomenon of central sensitization, in which injured and uninjured parts of the body exhibit lowered pain thresholds due to an alteration in central pain processing. it has furthermore been hypothesized that peripheral sources of nociception in the muscles may perpetuate central sensitization in chronic whiplash. the hypothesis explored in the present study was whether myofascial trigger points serve as a modulator of central sensitization in subjects with chronic neck pain.

**Design:**

controlled case series.

**Setting:**

outpatient chronic pain clinic.

**Subjects:**

seventeen patients with chronic and intractable neck pain and 10 healthy controls without complaints of neck pain.

**Intervention:**

symptomatic subjects received anesthetic infiltration of myofascial trigger points in the upper trapezius muscles and controls received the anesthetic in the thigh.

Outcome measures: pre and post injection cervical range of motion, pressure pain thresholds (ppt) over the infraspinatus, wrist extensor, and tibialis anterior muscles. sensitivity to light (photophobia) and subjects' perception of pain using a visual analog scale (vas) were also evaluated before and after injections. only the ppt was evaluated in the asymptomatic controls.

**Results:**

immediate (within 1 minute) alterations in cervical range of motion and pressure pain thresholds were observed following an average of 3.8 injections with 1–2 cc of 1% lidocaine into carefully identified trigger points. cervical range of motion increased by an average of 49% (p = 0.000) in flexion and 44% (p = 0.001) in extension, 47% (p = 0.000) and 28% (p < 0.016) in right and left lateral flexion, and a 27% (p = 0.002) and 45% (p = 0.000) in right and left rotation. ppt were found increased by 68% over the infraspinatus (p = 0.000), by 78% over the wrist extensors (p = 0.000), and by 64% over the tibialis anterior (p = 0.002). among 11 subjects with photophobia, only 2 remained sensitive to light after the trigger point injections (p = 0.033). average vas dropped by 57%, from 6.1 to 2.6 (p = 0.000). no significant changes in ppt were observed in the control group following lidocaine infiltration of the thigh.

**Conclusion:**

the present data suggest that myofascial trigger points serve to perpetuate lowered pain thresholds in uninjured tissues. additionally, it appears that lowered pain thresholds associated with central sensitization can be immediately reversed, even when associated with long standing chronic neck pain. although the effects resulting from anesthesia of trigger points in the present study were temporary, it is possible that surgical excision or ablation of the same trigger points may offer more permanent solutions for chronic neck pain patients. further study is needed to evaluate these and other options for such patients.

## Introduction

Chronic pain from injury, including injury from whiplash trauma, is associated with centrally mediated hyperalgesia, also known as central sensitization. [1] Several authors have described lowered pain thresholds in uninjured tissues, and explained the finding as the expression of an abnormal processing of nociceptive information in the brain and spinal cord. [2-7] Others have postulated that chronicposttraumatic myalgia (muscle pains) mayperpetuateandaccentuate thepain status of afflicted patients, [8,9] and Ge et al. recently reported experimental evidence of a physiologic link between myofascial trigger points and central sensitization in patients with shoulder pain of myofascial origin. [10] In contrast, Curatolo et al, in a study of chronic whiplash patients, reported that anesthetic infiltration of painful or tender points did not alter the signs of central sensitization. [11] An explanation for this discrepancy may lie in the definition of what constitutes a myofascial pain generator. While both 'tender points' and myofascial trigger points are painful to palpation, only myofascial trigger points will fasciculate or "twitch" when probed with a needle. [12] The presence of a twitch response to needle probing has been experimentally demonstrated as a necessary prerequisite for trigger point deactivation and pain relief with local anesthetic injection. [13] Based on these previously reported findings, it is reasonable to hypothesize that anesthetization of carefully identified trigger points may alter findings of central sensitization in patients with chronic neck pain.

In the present study the authors set out to evaluate whether anesthetic infiltration of myofascial trigger points in patients with chronic and refractory neck pain can affect pain thresholds in uninjured parts of the body.

## Methods

Seventeen participants were recruited from a group of twenty-threepatients who were referred for surgical evaluation for chronic and refractory neck pain. Inclusion criteria were as follows:

1. Male or female, 19–65 years of age.

2. Intrusive daily neck pain for at least 12 months.

3. Failure of conservative therapies, e.g. physical therapy, chiropractic manipulation, or acupuncture.

Exclusion criteria included signs or symptoms of radiculopathy or myelopathy, or radiographic evidence of significant spine pathology.

Institutional Review Board oversight and approval was provided by the Spinal Injury Foundation in Westminster, Colorado.

### Pre-intervention evaluations

1. Cervical range of motion (CROM) in flexion, extension, right and left lateral flexion, and right and left rotation was determined with inclinometry. This evaluation was only performed on the study subjects and not the controls and the subjects were blinded as to the results.

2. Pressure pain thresholds (PPT) were determined on one side of the body using algometry as described by Koelbaek-Johansen et al. [2] Laterality was determined according to subjects' indication of the side with the most intense symptoms. Left was chosen as the default side ifa patient could not differentiate one side as more symptomatic then the other. The calibrated algometer had a range of 0.5 – 25 lbs (1.1–13.4 kg) distributed over a 1 cm circular tip. Test sites were in three different muscles: infraspinatus – 3 cm inferior to the scapular spine and 3 cm lateral to the medial scapular border; wrist extensor – 5 cm distal to the lateral epicondyle with forearm in full supination; tibialis anterior – 5 cm distal to the tibial tuberosity, and 2 cm lateral to the anterior tibial margin. Pain thresholds were determined by slowly and gradually increasing the pressure of the algometer tip against the marked test site. Identical protocols were used to assess pain thresholds in the study group and the control group, and all subjects were blinded with to the values recorded from each test.

3. Presence or absence of photophobia was determined by shining light from an ophthalmoscope into the ipsilateral eye for 3 seconds. Photophobia was considered present if the subject confirmed immediate onset or worsening of periocular pain or headaches. This evaluation was only performed on the study subjects.

4. Subjective assessment of neck painwas madeusing a visual analogue scale (VAS) graded 0–10. This evaluation was only performed on the study subjects.

### Intervention

One examiner performed all interventions. Myofascial trigger points (TP) were mapped through palpation alongthe upper trapezius, and traced on the skin with a permanent marker. Next, the focal pain generator within each trigger point was identified through probing witha 25 gauge needleforsharp pain and a twitchresponse, and injected with 1–2 cc 1% lidocaine (10–20 mg). The procedure was repeated at other previously identified and marked TPs until the subject indicated a significant relief of neck pain. Aminimum of one, but never more than eight trigger points were injected in any of the subjects, and no single TP was injected more than once. The total amount of 1% lidocaine injected to any one subject varied from 2 cc to 10 cc.

### Post-intervention evaluation

1. All pre-injection measurements(PPT, CROM, Photophobia, and VAS) were repeated following the injections of lidocaine. Identical techniques were used, with the exception that PPTwas only evaluated once. Post-injection PPT was determined within 60 seconds of the final injection of local anesthetic (study group and controls), and remainingdata was collected immediately thereafter. On no instance did more than seven minutes elapse between the time of the final injection andcompletion of the data collection procedure.

### Control Group

In addition to the symptomatic subjects a group of 10 volunteers with no history of chronic neck pain was recruited to serve as a control for the intervention effects. The controls were each evaluated for PPT twice at the same three sites as the symptomatic subjects prior to an injection of 6 cc of1% lidocaine in the left thigh, and then re-evaluated for PPT within 1 minute of the injection. The purpose of the control group was to assess the effect of systemic lidocaine on PPT versus the more specific trigger point infiltration in the symptomatic subjects.

### Statistical analysis

Paired-sample t-tests were conducted on pre-injection/post-injection pairs for each of the ten numerical evaluation measures – six cervical range of motion types, three PPT sites, and the visual analog scale (Analyze-It, Leeds UK).

## Results

Of the 23 patients presenting for evaluation 17 fit the inclusion criteria. The 6 patients who were excluded either had chronic pain that was not in the neck or had neck pain for less than 12 months. The 17 remaining patients consisted of14 (82%) female and 3 (18%) male subjects ranging in age from 26 to 59, with a mean age of 42.4 (SD 9.7). The duration of symptoms ranged from 1.5 to 18 years, with a mean duration of 8.7 years (SD 6.0). All except one described a traumatic episode as the precipitating factor for their neck pain, and most were injured in a traffic crash. (Table 1) Six subjects described current or past suicidal ideationbecause ofunremitting pain. Although all subjects used non-steroidal anti-inflammatory drugson a regularbasis, none reportedmore than occasional opioid use. All of the patients had been diagnosed with a chronic pain syndrome that was intended to describe their unremitting symptoms of neck pain, including myofascial pain syndrome, fibromyalgia syndrome, or simply "chronic neck pain." None of the subjects had been diagnosed with radiculopathy or myelopathy or cervical central stenosis, although there were several with nonspecific diagnoses of cervical spondylosis.

**Table 1:** Attibuted cause of chronic neck pain

Attributed cause of chronic pain	# subjects (%)
Traffic crash	11 (65)

Low speed rear impact	3 (18)

Moderate speed rear impact	3 (18)

Side impact	3 (18)

Front impact	2 (12)

Skiing injury	3 (18)

Fall	1 (6)

Lifting	1 (6)

Insidious onset	1 (6)

**Total**	**17 (100)**

The control group consisted of 6 male and 4 female volunteers with a mean age of 39.6 (SD 12.1).

**Figure 1:**
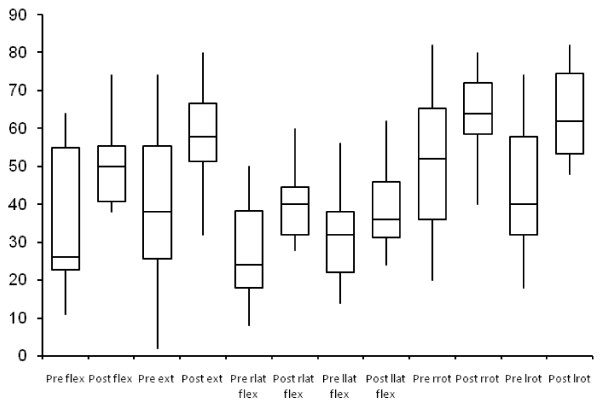
**Pre and post injection cervical range of motion in degrees**. The abbreviations are as follows: Flex – flexion, Ext – extension, rlat flex – right lateral flexion, llat flex – left lateral flexion, rrot – right rotation, lrot – left rotation.

As would be expected in a population of chronic neck pain patients, pre-injection CROMin the study group was generally lower thanstandard reference values. [14] Following the TP injection all subjects demonstrated an increase in CROM in all directions. (Figure 1; Table 2)

**Table 2:** Pre and post injection cervical range of motion

CROM (degrees)	Pre-injection (mean, SD)	Post-injection (mean, SD)	% change	p-value
Flexion	33.9, 17.7	50.5, 10.5	49	0.000

Extension	40.6, 20.6	58.4, 12.6	44	0.001

R Lat Flexion	27.2, 11.9	40.0, 8.9	47	0.000

L Lat Flexion	31.0, 12.7	39.6, 12.3	28	0.016

R Rotation	50.2, 19.1	63.7, 10.5	27	0.002

L Rotation	44.3, 15.6	64.1, 11.3	45	0.000

In the study group, there was no significant difference in pre-injection pain threshold values at the shoulder or forearm;readings for the second pre-injection tibialis anterior PPT test were significantly lower than for the first. (Table 3) In order to avoid selection bias in favor of significance, the more conservative (higher) first pre-injection test was used to establish the pre-injection PPT in tibialis anterior. The pooled mean of the two pre-tests was used to establish the pre-injection level of the other two test sites – infraspinatus and wrist extensor.

**Table 3:** Pre-injection PPT values, and pooled mean used for comparison with post-injection PPT

**PPT **(lbs)	Pre-injection 1 (mean, SD)	Pre-injection 2 (mean, SD)	p-value (1 vs. 2)	Pooled mean (mean, SD)
**Infraspinatus**	4.1, 2.1	3.8, 1.9	0.111	4.0, 2.0

**Wrist Ext**	4.3, 2.9	4.3, 2.6	0.986	4.3, 2.7

**Tib Ant**	8.2, 4.3	7.3, 3.9	0.003	7.7, 4.1

**Figure 2:**
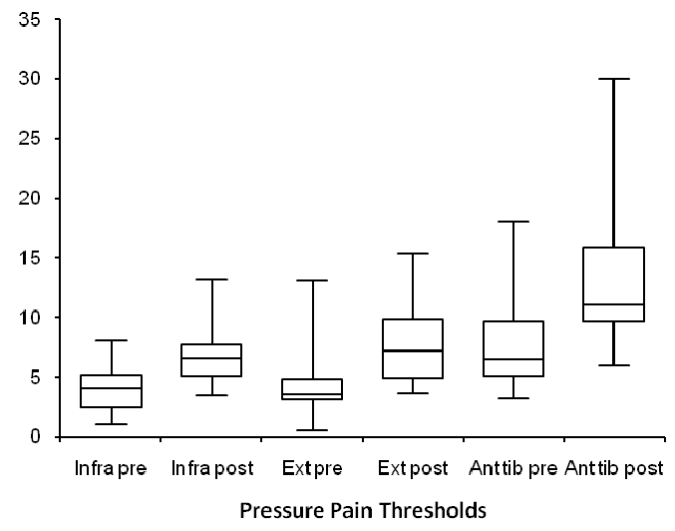
**Pre and post pressure pain thresholds as measured in pounds of pressure**. The abbreviations are as follows: infra – infraspinatus, Ext – wrist extensors, Ant tib – Anterior tibialis.

Statistically significant increases in pressure pain thresholds were documented within the symptomatic groupafter the TP injections within 1 minute. (Tables 3 and 4) Post-injection pressure pain thresholds were 68, 78, and 64% greater at the infraspinatus, wrist extensors, and tibialis anterior, respectively, in comparison with the pre-injection thresholds in the study group. (Figure 2).

**Table 4:** Pre and post injection changes in PPT, all measurements taken within 1 minute of the final injection

**PPT **(lbs)	Pre-injection (mean, SD)	Post-injection (mean, SD)	% change	p-value
**Infraspinatus**	4.0, 2.0	6.7, 2.3	68	0.000

**Wrist Ext**	4.3, 2.7	7.7, 3.4	78	0.000

**Tib Ant**	8.2, 4.3	13.4, 6.8	64	0.002

Among the controls there were no significant differences between the two pre-injection PPT values or between the second pre-injection PPT measurement and the post intramuscular (IM) thigh injection PPT values at any of the three evaluation sites. (Table 5)

**Table 5:** Pressure pain threshold for the controls

**PPT **(lbs)	Pre-injection 1 (mean, SD)	Pre-injection 2 (mean, SD)	p-value (1 vs. 2)	Post-injection (mean, SD)	p-value
**Infraspinatus**	11.7, 5.0	10.8, 4.8	0.548	11.1, 4.4	0.437

**Wrist Ext**	8.6. 3.6	8.2. 4.1	0.348	8.3. 4.1	0.447

**Tib Ant**	12.9, 5.4	12.7, 5.6	0.509	12.1, 4.4	0.288

Of 11 (65%) subjectswho showed evidence of photophobia before TP injections, only2 (12%)described any pain or discomfort from light at the post-injection testing (p = 0.03).

**Figure 3:**
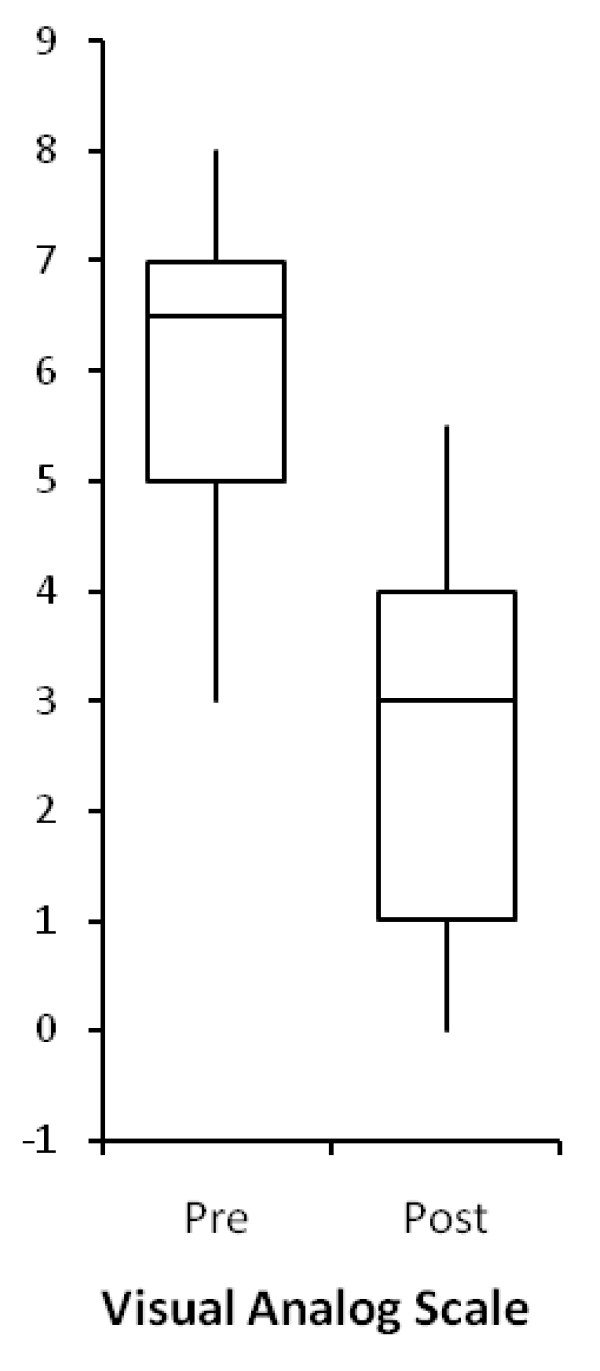
**Pre and post injection subjective pain level**.

A 57% reductionin neck pain was documented witha drop inVAS from a 6.1 (SD 1.5) before the trigger point injections, to 2.6 (SD 1.8) after the trigger point injections (p = < 0.001). (Figure 3). As the anesthetic wore off, however, all pre-injection symptoms returned to baseline over a matter of hours to several days.

## Discussion

The present data demonstrate a remarkably rapid change in central sensitization symptoms following the anesthetizing of painful trigger points. Since the infraspinatus PPT site was relatively close to areas that had been infiltrated with anesthetic, the wrist extensor and tibialis anterior pressure sites may be considered more reliable indicators of alterations in centralpain modulation within the study group; however, there were little differences among the three tested sitesand threshold increases were uniform.

Symptoms of light sensitivity (photophobia) resolved in all but 2 of the 11 subjects for the duration of the anesthetic. This result suggestsa central mechanism as the mediator between the myofascial trigger points andlight sensitivity; however, the phenomenon requires further investigation. The methods used in the present study were quite elementary; the outcome was simply the perception of eye or head discomfort when an opthalmoscope set on highest intensity was shined in the eye for 3 seconds. More precise methods of measuring eye discomfort thresholds vs. light intensity would have been required in order to draw any detailed quantitative conclusions. In contrast, the increase in cervical range-of-motion observed in the symptomatic cohort following the injections suggests that the initial finding of reduced motion was either a result of peripherally modulated pain inhibition (from the trigger points) or centrally mediated pain inhibition, or both.

Differences in the techniques adopted to identify and inject trigger points may explain why the present resultsdiffer from those of Curatolo et al, who found no changes in central sensitization with tender point (as opposed to trigger point) injections. Despite the difference in outcome between the present study and what was described by Curatolo et al., the hypothesis introduced by these authors, that central modulation of pain is maintained by continued peripheral nociception arising from painful muscles in the neck, provides a comprehensive explanatory model for our results. While physiological and anatomical characteristics of myofascial trigger pointsare not completely understood, it is reasonable to posit that these entities initially arise as a protective response to injury. Why such focal pain generators persist and remain active after a reasonable healing period for soft tissue injury remains unclear.

A nuisance effect that we attempted to control for was the potential impact of the infusion of intramuscular lidocaine on the pain thresholds of the symptomatic subjects. Prior authors have noted a decrease in experimentally induced hyperalgesia following intravenous (IV) administration of lidocaine. [15] Wu et al. also demonstrated a significant decrease in stump and phantom pain in an amputee population after 42 minutes of IV lidocaine infusion. [16] As opposed to the methods used in the present investigation, these studies involved IV infusions of lidocaine. Thus, the purpose of the control group in the present study was to evaluate the effect on pain thresholds of nonspecific IM lidocaine administration.

This is not to say that the present design is ideal; a randomized controlled trial, in which the controls were identical to the cases (both groups with chronic neck pain) would have been preferable. For this reason it is reasonable to interpret the results of the present study with a modicum of caution.

The most important conclusion to be drawn from our results, relate to the immediate and substantial changes in peripheral pain thresholds observed in the study group, where some participants had been symptomatic for more than two decades. It has been postulated that central sensitization is an expression of permanent structural or biochemical changes (neuroplasticity) in the central nervous system,[17] and therefore unlikely to change regardless of intervention Our findings therefore serve as an argument for central sensitization as a neuromodulatory process perpetuated by, and dependent upon peripheral sources of nociception referred to as trigger point. Rather than a neuroplastic condition, central sensitization may be a neuro *elastic *phenomenon. Our results also argue against psychological components as an etiological factor rather than consequence of chronic pain after whiplash injury. [18] While it has been suggested that litigation or monetary issues may augment chronic symptoms,[19] none of the subjects in the present study were involved in litigation and no reasonable interpretation of the present data allows for any attribution of the observations to financial motivation or emotional liability.

The number of subjects in the present study compares with previously published reports within the same field of investigation, in which the authors have presented data based on studies of 11–29 subjects. [1,3,5,6] Nonetheless, some degree of caution is warranted in extrapolating the results of this study to the general chronic neck pain population before further randomized and placebo-controlled trials help bolster the validity of the conclusions presented here. It is important to note that the success of the injections may largely depend upon the skill and experience of the operator [11].

It is reasonable to suggest, based upon our conclusions and those of prior authors, that algometry of both symptomatic and asymptomatic body sites may have a practical clinical application in the contemporary evaluation of treatment success in chronic neck pain patients.

Permanent solutions for the chronic pain conditions of the subjects in the present study are few; one suggestion is to surgically excise or ablate symptomatic trigger points that are associated with a decrease in local and generalized pain following anesthetization. [20] Such an approach, although intriguing, requires further description and study.

## Conclusion

Lowered pain thresholds related to chronic neck pain may rapidly reversed by precise location and anesthetization of trigger points. While the full implications of this finding are yet to be determined; treatments aimed at permanent ablation of peripheral pain generators may offer a means of long term relief for this patient population. The results of the present study refute claims that some or all of the pain experienced by chronic whiplash patients arises from psychosocial issues, as the only treatment experienced by the study subjects was directed at the identified trigger points. Although encouraging, further study is needed to explore the ramifications of these findings.

## Competing interests

The authors declare that they have no competing interests.

## Authors' contributions

MF: Research design, data collection, data analysis, writing and editing of manuscript. AN: Research design, study execution, writing and editing of manuscript. CC: Research design, editing of manuscript. All authors read and approved the final manuscript.
